# From diagnosis to disease-specific treatment: first experience with enzyme replacement therapy for Fabry disease in North Macedonia—a case series

**DOI:** 10.3389/fmed.2026.1857063

**Published:** 2026-05-29

**Authors:** Vlatko Karanfilovski, Igor G. Nikolov, Pavlina Dzekova Vidimliski, Svetlana Krstevska Balkanov, Galina Severova, Ana Stojanoska, Nikola Gjorgjievski

**Affiliations:** 1 University Clinic for Nephrology, Faculty of Medicine, Ss. Cyril and Methodius University in Skopje, Skopje, North Macedonia; 2 University Clinic for Hematology, Bone Marrow Transplantation Unit, Faculty of Medicine, Ss. Cyril and Methodius University in Skopje, Skopje, North Macedonia

**Keywords:** α-galactosidase, enzyme replacement therapy, Fabry disease, hypertrophy, left ventricular, kidney transplantation

## Abstract

**Background:**

Fabry disease is a rare X-linked lysosomal storage disorder caused by deficiency of α-galactosidase A, leading to progressive accumulation of globotriaosylceramide and Lyso-Gb3 across multiple organ systems. Timely initiation of enzyme replacement therapy (ERT) is critical to prevent irreversible organ damage; however, access to disease-specific treatment remains limited in many regions.

**Methods:**

We describe a prospective observational case series representing the first national experience with ERT in North Macedonia in two male patients with advanced Fabry disease following kidney transplantation. Clinical, biochemical, cardiac, neurological, and patient-reported outcomes were prospectively evaluated after initiation of agalsidase beta (1 mg/kg) and agalsidase alfa (0.2 mg/kg), respectively.

**Results:**

Both patients demonstrated substantial and sustained reductions in Lyso-Gb3 levels, confirming a robust biochemical response. Renal graft function remained stable without proteinuria, and no progression of cardiac involvement was observed. Clinical response varied between patients: the first patient experienced marked and sustained improvement in neuropathic pain and quality of life, whereas the second patient demonstrated persistent fluctuating neurological manifestations despite significant biochemical response. Persistent neurological impairment in the second patient was associated with combined central and peripheral nervous system involvement, including Fabry-related ischemic encephalopathy.

**Conclusion:**

In this two-patient case series, ERT was well-tolerated and associated with substantial reduction of biochemical disease burden and stabilization of renal graft and cardiac function. However, persistent neurological impairment despite marked Lyso-Gb3 reduction suggests limited reversibility of advanced central nervous system involvement, highlighting the importance of early diagnosis, family screening, and timely initiation of disease-specific therapy in Fabry disease.

## 1 Introduction

Fabry disease (FD, OMIM#301500) is a rare X-linked lysosomal storage disorder caused by deficiency of α-galactosidase A (GLA, OMIM*300644), resulting in progressive accumulation of globotriaosylceramide (Gb3) and its deacylated derivative Lyso-Gb3 within lysosomes of vascular endothelial cells, podocytes, cardiomyocytes, and neurons (1). Substrate accumulation triggers endothelial dysfunction, chronic inflammation, oxidative stress, and progressive fibrosis, ultimately leading to irreversible renal, cardiac, and cerebrovascular damage (2, 3). Clinically, classical FD presents with acroparesthesias, gastrointestinal symptoms, hypohidrosis, and angiokeratomas in childhood or adolescence, followed by progressive chronic kidney disease, hypertrophic cardiomyopathy, and ischemic stroke in adulthood (1). According to ClinVar Miner (4), more than 1,000 pathogenic and likely pathogenic mutation in the GLA gene have been reported contributing to the clinical variability in FD (4).

Enzyme replacement therapy (ERT) with recombinant α-galactosidase A (agalsidase alfa, agalsidase beta or pegunigalsidase alfa) represents the cornerstone of disease-specific treatment. By restoring enzymatic activity, ERT reduces intracellular Gb3 and circulating Lyso-Gb3 levels, attenuates substrate-mediated cellular toxicity, and aims to stabilize organ function (5–7). Clinical trials and long-term registry data have demonstrated that ERT slows the progression of renal and cardiac disease, reduces neuropathic pain, and improves health-related quality of life, particularly when initiated before advanced fibrosis and structural organ damage develop (3, 5, 7). However, therapeutic benefit is strongly influenced by disease stage at initiation, and reversibility of established organ injury remains limited.

**FIGURE 1:**
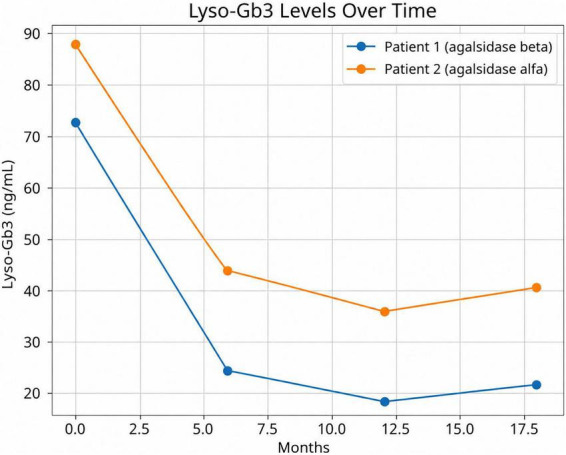
Longitudinal changes in Lyso-Gb3 levels during follow-up in Patient 1 treated with agalsidase beta and Patient 2 treated with agalsidase alfa.

Although Fabry disease was only recently recognized and reported in North Macedonia (1), access to disease-specific therapy has become available only in the past 18 months. The present study describes the first national experience with ERT for Fabry disease in North Macedonia and explores longitudinal clinical and biochemical outcomes in two patients with advanced multisystem disease. Longitudinal trends in Lyso-Gb3 levels during follow-up for both patients are shown in Figure 1.

## 2 Methods

This study represents a prospective observational case series describing the first national experience with ERT for Fabry disease in North Macedonia. Two adult male patients with genetically and biochemically confirmed Fabry disease who had previously undergone kidney transplantation were included. Both patients initiated ERT in July 2024 and were followed at the University Clinic for Nephrology, Skopje, according to the recommendations of the European Fabry Working Group and the national evidence-based guidelines for diagnosis, treatment, and follow-up of patients with Fabry disease (7, 8). Patient 1 received agalsidase beta (1 mg/kg every 2 weeks), while Patient 2 received agalsidase alfa (0.2 mg/kg every 2 weeks). The selection of ERT regimen was determined primarily by national drug availability, reimbursement procedures, and treatment regulations established by the Ministry of Health of the Republic of North Macedonia. Follow-up assessments included serial evaluation of plasma Lyso-Gb3 levels, renal graft function, proteinuria, cardiac imaging, neurological assessment, and patient-reported outcomes using the Brief Pain Inventory (BPI) and EQ-5D questionnaire (8). Lyso-Gb3 levels were measured using tandem mass spectrometry from dried blood spot samples at ARHIMEDlife^®^, a commercial diagnostic laboratory accredited according to the ISO 15189 standard.

## 3 Case presentation

### 3.1 Patient 1—agalsidase beta (fabrazyme^®^)

A 45-year-old male was diagnosed with Fabry disease during evaluation for advanced chronic kidney disease (CKD). A living-related kidney transplantation from his sister was successfully performed in 2023, 2 months after the diagnosis. His clinical history included long-standing acroparesthesias, intermittent abdominal pain, and intolerance to heat and cold. Diagnostic testing demonstrated undetectable α-galactosidase A activity of 0.1 μmol/L/h (cut-off value > 2.8 μmol/L/h), markedly elevated Lyso-Gb3, and a pathogenic hemizygous GLA variant c.443G > A (p.Ser148Asn). Baseline cardiac magnetic resonance imaging revealed concentric left ventricular hypertrophy with inferolateral myocardial edema and mesocardial fibrosis, consistent with Fabry-related cardiac involvement, with a moderately reduced left ventricular ejection fraction (LVEF 43.7%). ERT with agalsidase beta was initiated in July 2024. No infusion-related adverse events were observed during follow-up. After only a few infusions, the patient reported a marked reduction in peripheral neuropathic pain and improved tolerance to temperature changes. At baseline, neuropathic pain was of moderate intensity, with a Brief Pain Inventory (BPI) pain severity score of approximately 4.8 and a pain interference score of 4.5; following initiation of ERT, both parameters showed a progressive and sustained improvement, decreasing to approximately 3.0 and 2.8 at 6 months, 2.0 and 1.8 at 12 months, and 1.5 and 1.2 at 18 months, respectively. EQ-5D assessment demonstrated clinically meaningful improvement after ERT initiation. A robust biochemical response was observed, with Lyso-Gb3 decreasing from 73.4 ng/mL at baseline to 24.4 ng/mL at 6 months (67% reduction) and further to 18 ng/mL at 12 months (75.4% reduction). The patient maintained stable renal graft function without proteinuria under standard immunosuppressive therapy (cyclosporine, prednisolone and mycophenolic acid). During follow-up, no progression of cardiac involvement was observed on serial echocardiographic evaluations, and no new neurological or cerebrovascular events were recorded. Laboratory follow-up findings are summarized in Table 1.

**TABLE 1:** Laboratory findings of the patient treated with agalsidase beta before and after initiation of enzyme replacement therapy (ERT).

	Before ERT	3 months	6 months	12 months	18 months
Hemoglobin (120–180 g/L)	151	170	159	171	173
Red blood cells (4.2–5.5 × 10^12^/L)	5.13	5.3	5.4	5.9	5.8
White blood cells (4–9 × 10^9^/L)	10.4	12.7	11.2	13.1	13.4
Platelets (150–450 × 10^9^/L)	247	279	256	310	293
Albumin (35–50 g/L)	45	48	47	47	48
Proteinuria (<0.2 g/l)	0.05	–	0.18	0.05	0.054
Lactate dehydrogenase (<248 U/L)	444	270	242	403	321
Troponin (<34.2 ng/l)	–	114.5	110	100	**–**
Alanine aminotransferase (17–56 U/L)	19	25	20	20	22
Ca (2.1–2.6 mmol/L)	2.31	2.3	2.3	2.4	2.4
K (3.8–5.5 mmol/l)	5.32	4.4	4.4	4.6	4.7
Na (136–145 mmol/L)	140	144	139	137	147
Phosphate (0.8–1.4 mmol/L)	0.97	1.06	0.9	1	1
Creatinine (45–109 μmol/l)	133	127	122	119	118
eGFR (Cockcroft–Gault)	63.7	66.7	69.4	72	72
Lyso Gb3 (<3.5 ng/ml)	73.3	–	24.4	18	21.4

### 3.2 Patient 2—agalsidase alfa (replagal^®^)

A 44-year-old male was diagnosed with Fabry disease through family screening (cousin of the first patient). He had undergone living-related kidney transplantation from his wife 5 years prior to diagnosis for presumed primary glomerulopathy. His clinical presentation included anhidrosis, gastrointestinal symptoms, cardiac involvement, and progressive neurological manifestations. Additionally, the patient reported acroparesthesias and marked heat and cold intolerance since childhood. Two years prior to Fabry disease diagnosis, he had also been treated with escitalopram and vitamin therapy for mood disturbances and peripheral neuropathy. Diagnostic evaluation revealed undetectable α-galactosidase A activity and markedly elevated Lyso-Gb3 levels of 88.4 ng/mL (cut-off value 0.0–3.5 ng/mL). Baseline cardiac assessment demonstrated marked concentric left ventricular hypertrophy with preserved systolic function, confirmed by echocardiography and cardiac magnetic resonance imaging, consistent with Fabry-related cardiomyopathy. ERT with agalsidase alfa was initiated in July 2024. No infusion-related adverse events were observed during follow-up. Early during treatment, the patient reported substantial improvement in gastrointestinal symptoms and abdominal discomfort, accompanied by a significant biochemical response, with Lyso-Gb3 decreasing from 88.4 ng/mL at baseline to 44.3 ng/mL at 6 months (50% reduction) and to 35.6 ng/mL at 12 months (59.5% reduction). Despite optimization of symptomatic neurological therapy, including neuropathic pain modulators and supportive treatment, neurological involvement showed only partial and transient improvement. In January 2025, the patient developed peripheral facial nerve palsy, which improved clinically within one month. However, during follow-up, he continued to experience intermittent dysarthria, episodic muscle weakness, and reduced exercise tolerance, with fluctuating symptom intensity. Comprehensive neurological evaluation revealed combined central and peripheral nervous system involvement. Electromyography demonstrated an axonopathic polyneuropathy, mild in the upper extremities and moderate to severe in the lower extremities, with superimposed demyelinating features, predominantly affecting motor fibers. Visual evoked potentials showed a severe bilateral conduction defect, while somatosensory evoked potentials from the median nerve demonstrated bilateral impairment of central somatosensory pathways, with preserved tibial nerve responses. Brainstem auditory evoked potentials were within normal limits. Brain magnetic resonance imaging revealed multiple bilateral T2- and FLAIR-hyperintense lesions located in the deep and periventricular white matter, juxtacortical regions, pons, cerebellum, and splenium of the corpus callosum, consistent with Fabry disease–related ischemic encephalopathy. Several lesions demonstrated true diffusion restriction, supporting the presence of acute ischemic microinfarctions, without pathological contrast enhancement. Throughout follow-up, renal graft function remained stable without proteinuria under standard immunosuppressive therapy (cyclosporine, prednisolone and mycophenolic acid). Serial cardiac assessments showed no progression of systolic dysfunction, while neurological symptoms remained fluctuating, requiring continued neurological follow-up. Laboratory follow-up findings are summarized in Table 2.

**TABLE 2:** Laboratory findings of the patient treated with agalsidase alfa before and after initiation of enzyme replacement therapy (ERT).

	Before ERT	3 months	6 months	12 months	18 months
Hemoglobin (120–180 g/L)	178	181	163	177	179
Red blood cells (4.2–5.5 × 10^12^/L)	6	6.1	5.4	6	6
White blood cells (4–9 × 10^9^/L)	11.6	10.4	11.2	14	12.6
Platelets (150–450 × 10^9^/L)	256	252	256	277	190
Albumin (35–50 g/L)	49	46	47	47	51
Proteinuria (<0.2 g/l)	–	0.04	0.1	–	0.048
Lactate dehydrogenase (<248 U/L)	233	279	242	287	380
Troponin (<34.2 ng/l)	–	46.4	41.2	–	–
Aspartate Aminotransferase (10–40 U/L)	12	14	21	22	21
Alanine aminotransferase (17–56 U/L)	12	13	20	13	14
Ca (2.1–2.6 mmol/L)	2.6	2.4	2.4	2.5	2.5
K (3.8–5.5 mmol/l)	4.5	4.5	4.2	4.6	4.1
Na (136–145 mmol/L)	133	140	135	138	140
Phosphate (0.8–1.4 mmol/L)	1.02	1.06	0.8	0.8	0.9
Creatinine (45–109 μmol/l)	117	93	97	89	106
eGFR (Cockcroft–Gault)	54.3	59.3	65.4	68	61
Lyso Gb3 (<3.5 ng/ml)	88	–	44.3	35.6	40.6

A comparative overview of baseline clinical characteristics, organ involvement, treatment, and follow-up outcomes in both patients is presented in Table 3.

**TABLE 3:** Comparative overview of baseline clinical characteristics, organ involvement, treatment, and follow-up outcomes in both patients with Fabry disease treated with enzyme replacement therapy (ERT).

Parameter	Patient 1	Patient 2
Sex	Male	Male
Year of birth	1977	1978
Family relation	Index patient	Cousin of Patient 1
Genetic mutation	Pathogenic hemizygous GLA variant c.443G > A (p.Ser148Asn)	Pathogenic hemizygous GLA variant c.443G > A (p.Ser148Asn)
Fabry disease diagnosis	May 2023	September/October 2023
CKD stage 5	Yes	Yes
Hemodialysis before transplantation	6 months	Approximately 5 years
Kidney transplantation	June/July 2023	2018
Donor type	Living-related donor (sister)	Living-related donor (wife)
α-galactosidase A activity	Undetectable	Undetectable
Baseline Lyso-Gb3	73.4 ng/mL	88.4 ng/mL
Cardiac involvement	Fabry cardiomyopathy with left ventricular hypertrophy	Fabry cardiomyopathy with left ventricular hypertrophy
Neurological involvement	Mild/stable symptoms	Significant central and peripheral nervous system involvement
Brain MRI findings	No significant abnormalities	Extensive ischemic white matter lesions with acute microinfarctions
ERT regimen	Agalsidase beta	Agalsidase alfa
ERT initiation	July 2024	July 2024
Biochemical response	Significant reduction in Lyso-Gb3	Significant reduction in Lyso-Gb3
Renal outcome during follow-up	Stable graft function without proteinuria	Stable graft function without proteinuria
Cardiac outcome during follow-up	Stable cardiac status	Stable cardiac status
Neurological outcome during follow-up	Clinically stable	Partial improvement with persistent fluctuating symptoms
Infusion-related adverse events	None	None

## 4 Discussion

This report presents the first real-world experience with ERT for Fabry disease in North Macedonia and illustrates the multisystemic effects of both drugs in two male patients with advanced organ involvement, including renal transplantation, cardiomyopathy, and neurological manifestations. Fabry disease is characterized by progressive accumulation of globotriaosylceramide and related metabolites, leading to irreversible organ damage if untreated (3). The primary aims of ERT are reduction of substrate accumulation, stabilization of organ function, and improvement of health-related quality of life (3). The greatest benefit is achieved when therapy is initiated early; however, as demonstrated by both patients in this series, delayed diagnosis remains common in real-world practice, particularly in regions without structured screening programs.

Renal involvement remains a major determinant of long-term prognosis in Fabry disease, and the effect of ERT on kidney outcomes is strongly dependent on disease stage at treatment initiation. Large systematic reviews and meta-analyses demonstrate that ERT is most effective in stabilizing renal function when initiated before the development of significant proteinuria and advanced glomerulosclerosis (9). Baseline proteinuria has consistently emerged as one of the strongest predictors of renal trajectory, with patients presenting with higher urinary protein excretion experiencing faster eGFR decline despite treatment. Recent longitudinal cohort data from a national Fabry center further demonstrated that older age at diagnosis, higher albuminuria, and pre-existing cardiovascular disease are associated with accelerated renal decline, progression to kidney replacement therapy, and increased long-term mortality (10). Experimental studies additionally show that Lyso-Gb3 exerts direct toxic effects on podocytes, mesangial cells, and tubular epithelial cells, promoting inflammation, fibrosis, and progressive nephron loss (2). Despite its clear pathogenic role, current evidence does not support Lyso-Gb3 as a reliable biomarker for monitoring renal disease progression, particularly in advanced disease or post-transplant settings. In our cohort, both patients had undergone kidney transplantation prior to ERT initiation, representing advanced Fabry nephropathy. Consistent with registry and real-world observational data, ERT was associated with stable graft function and absence of proteinuria during follow-up, although established renal damage was not reversible (6).

Cardiac involvement represents a major cause of morbidity and mortality in Fabry disease. Large real-world cohorts have demonstrated a substantial burden of arrhythmias, atrial fibrillation, ventricular tachycardia, heart failure, and cerebrovascular events, particularly among male patients with advanced disease and higher baseline disease severity scores (11). ERT can stabilize left ventricular mass, wall thickness, and systolic function, particularly when initiated before the development of extensive myocardial fibrosis (5). Cardiac magnetic resonance imaging studies have shown that while ERT may attenuate hypertrophic remodeling, its ability to reverse established fibrosis is limited. The presence of late gadolinium enhancement, especially in the inferolateral wall, reflects irreversible myocardial injury and is associated with reduced therapeutic response (3). Both patients in our series demonstrated advanced hypertrophic cardiomyopathy at baseline. During follow-up, ERT was associated with clinical and echocardiographic stability without progression of systolic dysfunction or chamber dilation, findings consistent with real-world evidence suggesting that ERT primarily slows cardiac disease progression rather than inducing regression once fibrotic remodeling has occurred (6).

Neurological involvement is one of the most challenging aspects of Fabry disease management. Real-world evidence indicates that ERT has a differential impact on the peripheral and central nervous systems, with more consistent benefits observed in peripheral neuropathy than in central nervous system (CNS) involvement (12, 13). Peripheral nervous system manifestations, particularly small-fiber neuropathic pain, show clinically meaningful but typically incomplete improvement following ERT initiation, with reductions in pain severity and pain interference frequently reported. However, complete symptom resolution is uncommon, and adjunctive symptomatic therapy is often required (12, 13). In contrast, the effect of ERT on central nervous system manifestations is limited. Due to limited blood–brain barrier penetration, ERT does not reverse established white-matter lesions or ischemic CNS injury, although vascular stabilization may occur (12, 14).

In both patients, ERT resulted in marked and sustained reductions in Lyso-Gb3 levels, confirming its role as a sensitive pharmacodynamic marker of biochemical response. However, our observations also underscore that Lyso-Gb3 reduction does not uniformly translate into parallel clinical improvement across all organ systems, particularly in advanced disease stages. While the first patient demonstrated overall clinical stabilization, the second patient—despite significant biochemical response—experienced persistent neurological impairment with fluctuating dysarthria, episodic muscle weakness, and reduced exercise tolerance. Neurophysiological and neuroimaging studies demonstrated combined peripheral and central nervous system involvement, including Fabry-related ischemic white matter changes. Recent large real-world cohort studies similarly demonstrate persistent cerebrovascular, cardiovascular, and multisystem morbidity despite disease-specific therapy, particularly in male patients with advanced disease and higher baseline severity scores (10, 11). These findings suggest that Lyso-Gb3 is best interpreted as a biomarker of biochemical substrate reduction rather than a direct indicator of organ-specific clinical reversibility. The second patient presented with substantially higher disease burden at baseline, including prolonged dialysis exposure prior to transplantation and more pronounced neurological and cardiac involvement, likely reflecting irreversible structural CNS and cardiac injury prior to ERT initiation. This dissociation between biochemical response and persistent neurological impairment also raises questions regarding treatment optimization. Agalsidase alfa and agalsidase beta differ primarily in dosing, with recommended regimens of 0.2 and 1 mg/kg every 2 weeks, respectively, and some studies suggest greater biochemical substrate reduction with higher-dose therapy in selected patients with high disease burden (15, 16). Switching from agalsidase alfa to higher-dose agalsidase beta has been associated with attenuation of eGFR decline and further reduction of Lyso-Gb3 levels in patients with suboptimal response or disease progression on prior therapy (15). However, available evidence indicates that such strategies primarily affect biochemical and renal parameters, while cardiac and neurological outcomes tend to remain stable rather than substantially improve (15, 16). Therefore, therapeutic decisions should be individualized and guided by overall disease trajectory and long-term treatment goals. Meaningful comparison between both ERT regimens cannot be made in a two-patient observational series.

This study has several limitations. First, the sample size was very small, reflecting the rarity of Fabry disease and the recent introduction of enzyme replacement therapy in North Macedonia, which limits the generalizability of the findings. Second, the absence of a comparator group precludes formal evaluation of treatment efficacy. Third, both patients initiated therapy at an advanced stage of multisystem disease following kidney transplantation, which likely limited the reversibility of organ involvement, particularly neurological manifestations. Finally, although follow-up was prospective, the observation period remains relatively short for a lifelong progressive disorder such as Fabry disease.

In conclusion, this report describes the first real-world experience with ERT for Fabry disease in North Macedonia. In these two patients with advanced multisystem disease, ERT was well-tolerated and associated with substantial reductions in Lyso-Gb3 levels, stable renal graft function, and cardiac disease stabilization. However, persistent neurological impairment despite biochemical response—particularly in the patient with established central nervous system involvement—highlights the limited reversibility of advanced neurological damage. Although interpretation is inherently limited by the small sample size and observational design, these findings further support the importance of early diagnosis, family screening, and timely initiation of disease-specific therapy in Fabry disease.

## Data Availability

The datasets presented in this study can be found in online repositories. The names of the repository/repositories and accession number(s) can be found in the article/supplementary material.
